# Investigation of Copper Alloying in a TNTZ-Cu_x_ Alloy

**DOI:** 10.3390/ma12223691

**Published:** 2019-11-08

**Authors:** Lee Fowler, Arno Janse Van Vuuren, William Goosen, Håkan Engqvist, Caroline Öhman-Mägi, Susanne Norgren

**Affiliations:** 1 Division of Applied Material Science, Department of Engineering Sciences, The Ångström Laboratory, Uppsala University, Box 534, 75121 Uppsala, Sweden; lee.fowler@angstrom.uu.se (L.F.); hakan.engqvist@angstrom.uu.se (H.E.); susanne.norgren@angstrom.uu.se (S.N.); 2 Centre for High Resolution Transmission Electron Microscopy, Department of Physics, Nelson Mandela University, 6031 Port Elizabeth, South Africa; arno.jansevanvuuren@mandela.ac.za (A.J.V.V.); william.goosen@mandela.ac.za (W.G.); 3 Sandvik, Lerkrogsvägen 13, 12680 Stockholm, Sweden

**Keywords:** titanium alloy, microstructures, biomaterial, TNTZ

## Abstract

Alloying copper into pure titanium has recently allowed the development of antibacterial alloys. The alloying of biocompatible elements (Nb, Ta and Zr) into pure titanium has also achieved higher strengths for a new alloy of Ti-1.6 wt.% Nb-10 wt.% Ta-1.7 wt.% Zr (TNTZ), where strength was closer to Ti-6Al-4V and higher than grade 4 titanium. In the present study, as a first step towards development of a novel antibacterial material with higher strength, the existing TNTZ was alloyed with copper to investigate the resultant microstructural changes and properties. The initial design and modelling of the alloy system was performed using the calculation of phase diagrams (CALPHAD) methods, to predict the phase transformations in the alloy. Following predictions, the alloys were produced using arc melting with appropriate heat treatments. The alloys were characterized using energy dispersive X-ray spectroscopy in scanning transmission electron microscopy (STEM-EDS) with transmission Kikuchi diffraction (TKD). The manufactured alloys had a three-phased crystal structure that was found in the alloys with 3 wt.% Cu and higher, in line with the modelled alloy predictions. The phases included the α-Ti (HCP-Ti) with some Ta present in the crystal, Ti_2_Cu, and a bright phase with Ti, Cu and Ta in the crystal. The Ti_2_Cu crystals tended to precipitate in the grain boundaries of the α-Ti phase and bright phase. The hardness of the alloys increased with increased Cu addition, as did the presence of the Ti_2_Cu phase. Further studies to optimize the alloy could result in a suitable material for dental implants.

## 1. Introduction

Commercially pure titanium (CP-Ti) and the Ti-6Al-4V (Ti-64) alloy are standard materials for medical implants, but problems with both have come to light with in vivo use. The former has a lower yield strength, which for grades 1–4, ranges from 170–480 MPa, respectively [1]. The latter contains vanadium which could be toxic [2] and aluminium which has been linked to Alzheimer’s disease [3]. These problems have inspired development of β-Ti [4,5,6,7] and α + β-Ti [8] alloys to replace these. Of the novel alloys to date, the Ti-Nb-Ta-Zr (TNTZ) system has shown promising properties for biocompatibility and strength comparable to Ti-64 [9,10,11]. Depending on alloying compositions, this alloy can be manipulated to have Young’s moduli lower than Ti-64, which is an appropriate step towards achieving elasticity similar to cortical bone in future [12]. All these Ti-alloys are however vulnerable to biofilm formation and patients could require antibiotic treatments in the event of infection [13,14]. In lieu of the growing problem with antibiotic resistance [15], antibacterial alloys could be useful for the biomaterials field. 

Excessive addition of Cu in Ti-Cu_x_ binary alloys could however lead to toxicity [13] or material embrittlement, which is a disadvantage for load bearing biomaterials [16]. Therefore careful microstructural design is required so that the mechanical properties can be optimized for the intended application. While these findings are descriptive of a binary alloy of Ti-Cu_x_, and Cu alloying has been performed on Ti-13Nb-13Zr-10Cu [17], it is envisioned that a similar antibacterial ability may be engineered into other quaternary alloys, i.e. TNTZ [11]. 

The alloying of Cu to TNTZ could lead to a novel alloy with several advantages, but microstructural, mechanical and biological properties still require careful study for optimization. Binary systems such as Ti-Cu show clear microstructural dependence on crystal relationships, chemical phases present, chemical-migration and -ordering [18]. Donthula et al. [18] and Contieri et al. [19] in particular have described the actively driven eutectoid transformation of β-Ti to α-Ti and Ti_2_Cu, which elucidates why β-Ti is not found in rapidly quenched alloys of this variety [20]. In contrast, studies on TNTZ without Cu present, show β-Ti and α-Ti microstructure with metastable β-phases present [21]. These two alloy systems are micro-structurally dissimilar, which further motivates the investigation into Cu addition in the TNTZ alloy systems. For these reasons the present study aims to determine the effects of Cu addition to an existing alloy of TNTZ [11], and characterize the material.

## 2. Materials and Methods 

### 2.1. Computational Modelling of Alloys

The impact of Cu to the Ti-Nb-Ta-Zr system was modelled using computational thermodynamic modelling based on the CALPHAD approach [22], using the Thermo-Calc software (Thermo-Calc software AB, Solna, Sweden) and the SSOL5 database, available from www.thermocalc.se. 

Since the TNTZ alloy [11] has low additions of Zr and Nb, it is hypothesized that these will remain in the solid solution of the α and β phases, respectively. The Cu, being a, β-eutectoid stabilizer, is also expected to create a eutectoid microstructure of lathes, but with increasing Cu additions, a Cu-rich phase is predicted to precipitate. It is likely that this phase will precipitate preferentially at the grain boundaries, which could lead to embrittlement. In the literature there are two contradictory predictions of the Ti-Cu phase diagram where the first Cu-rich phase is either Ti_2_Cu [23] or Ti_3_Cu [24]. The thermodynamic modelling done in this work is based on the first description [15], which excludes Ti_3_Cu which is a metastable phase, based on the observations by Zhang et al. [25]. Therefore, the binary Ti-Cu system was taken from the 1996 description by Kumar et al. [23].

**Figure 1:**
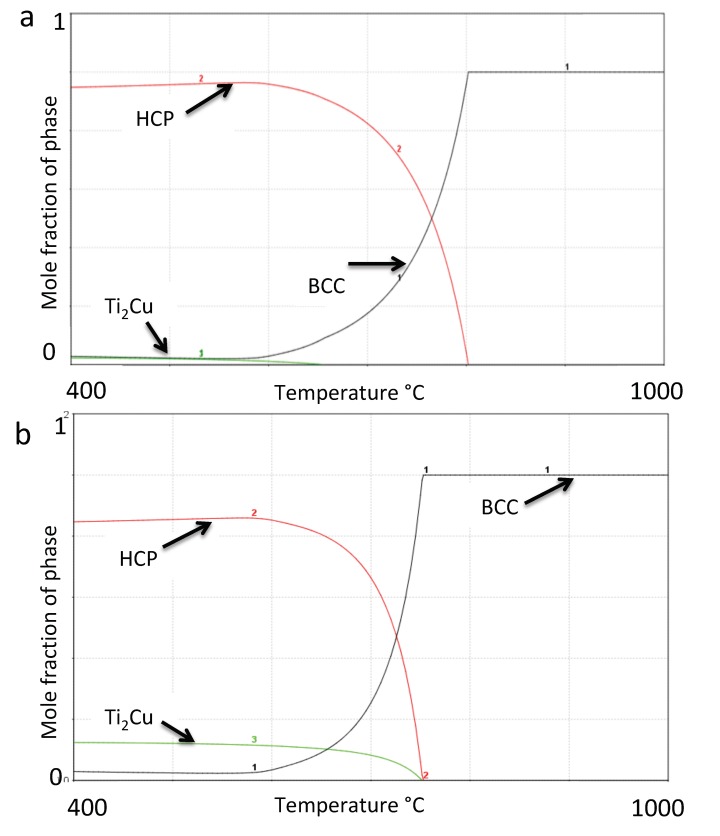
Mole fraction of phases as a function of temperature for (**a**) the Ti-Nb-Ta-Zr-1 wt.% Cu alloy and (**b**) the Ti-Nb-Ta-Zr-5 wt.% Cu alloy. Composition of the alloys can be found in Table 3.

The results should be regarded as an initial prediction of the phases and transition temperatures. This is due to the fact that the Ta-Cu binary, the Ta-Nb-Cu ternary and the Ti-Ta-Cu ternary systems have not been thermodynamically assessed and thus are lacking in the SSOL5 database. Nevertheless, the predictions given by the calculations are useful as a starting point for alloy development and to guide the experimental work. The Ti-1.7 wt.% Nb-10.1 wt.% Ta-1.6 wt.% Zr (TNTZ) has been modelled previously [11] and gave only α and β phases. The equilibrium phases as a function of temperature were modelled for this alloy with increasing Cu additions (0 wt.% Cu, 1 wt.% Cu, 3 wt.% Cu, 5 wt.% Cu and 10 wt.% Cu). In Figure 1a,b the phase fractions in the alloys with 1 wt.% Cu and 5 wt.% Cu addition are shown. Given the prerequisites mentioned, the Ti_2_Cu forms at 656 °C in the 1 wt.% Cu alloy (Figure 1a) in thermodynamic equilibrium; However, since the mole fraction is very low it is not likely to nucleate due to kinetic reasons. Nevertheless, when the phase fraction of Ti_2_Cu increases with Cu addition, already at 3 wt.% Cu and here at 5 wt.% Cu (Figure 1b), the phase fraction is considerable. The modelling resulted in the phase transition temperatures given in Table 1, where transus in this case, is the temperature above which the phase is no longer stable.

The wt.% Cu added to each alloy introduces a change in the Gibb’s free energy for each of the predicted phases [26], and depending on the resultant driving force ($\Delta G_{v}$), the phase development will proceed as specified according to phase reactions (Table 2). The cooling rate will furthermore determine the microstructure, where metastable martensitic phases (α’ and ω) could form and change the resultant material properties during a rapid quenching.

### 2.2. Production of Alloys

Alloys of Ti-Nb-Ta-Zr-Cu_x_ were produced in the range from 0 to 10 wt.% Cu (Table 3). Pre-alloyed Ti-Nb-Ta-Zr (Sandvik AB, Stockholm, Sweden) and 99.9999% pure copper rods (365327-21.5G, Sigma Aldrich, MO, USA) were used to produce the investigated alloys. Alloys were re-melted 5 times in an arc furnace, then melted into rods in the same furnace (Series 5 Bell Jar, Centorr Vacuum industries, Nashua, NH, USA). Partial homogenisation was achieved by the turning-over of the melted alloys between each of the five melting events. Complete homogenisation was achieved by heat treatments of the alloys at 988 °C, which is above all the calculated β-transus temperatures, for 48 h, then 747 °C for 18 h followed by a rapid quench. The second temperature was chosen based on the solution treatment for the Ti_2_Cu. The annealing was done in vacuumed ampoules, at a pressure of 1.333 mbar, to reduce the oxygen content in the alloys. All alloys were quenched in salt brine water. Thereafter all samples were embedded in Bakelite resin (PolyFast, Stuers, Ballerup, Denmark) and cut into slices using an aluminium oxide disk (50A13, Struers) before further analysis. Metallographic preparation included grinding according to the three-step preparation developed by Vander Voort [27], which was appropriately adapted (Table 4).

### 2.3. Calorimetric Measurements of Phase Transformations 

The β-transus and phase transformation temperatures were measured by differential scanning calorimetry (DSC) using a Netzsch STA 409 CD (NETZSCH-Gerätebau GmbH, Selb, Germany). Aluminium oxide crucibles were used. The 1, 3, 5 and 10 wt.% Cu samples were chosen for these measurements, which have gone through the above-mentioned heat treatments with rapid cooling. The rate of the temperature change was 10 °C/min. The phase transformation temperatures were determined using the onset method.

### 2.4. X-ray Diffraction

X-ray diffraction patterns were recorded in the Bragg-Brentano geometry using a Bruker TWIN-TWIN diffractometer (D8 Advance, AXS GmbH, Karlsruhe, Germany) with Ni-filtered Cu Kα radiation (Kα1 = 1.540598 Å). Samples were polished to 6 µm using a diamond suspension (DiaDuo-2, Struers). Crystalline phases were studied in EVA software version 4.3 (Bruker, Billerica, MA, USA). The identified phases from the ICDD database PDF–4+ 2019 [28] included PDF# 04-003-1382 (Ti_2_Cu), PDF# 00-044-1294 (HCP-Ti) and PDF# 03-065-9616 (HCP Ti-Ta).

### 2.5. Microstructural Studies 

The microstructure of the samples was studied in scanning electron microscopy (SEM), focused ion beam (FIB) and scanning transmission electron microscopy (STEM) using a Zeiss Merlin (Oberkochen, Germany), an FEI Helios Nano-Lab (Brno, Czech Republic), and a JEOL 2100 TEM/STEM (Tokyo, Japan), respectively.

The Zeiss SEM and JEOL TEM/STEM were equipped with INCA AZtec Energy Dispersive X-ray Spectroscopy systems (EDS, Oxford Instruments, High Wycombe, UK) while the SEM and FIB-SEM each were equipped with back scatter, in-lens and Everhart-Thornley detectors. The JEOL TEM/STEM was additionally equipped with an annular dark field and bright field detector (JEOL, Tokyo, Japan).

For crystallographic investigations, Transmission Kikuchi Diffraction (TKD) was done with a custom made sample holder, in a JEOL 7001F SEM instrument (JEOL, Tokyo, Japan) equipped with a Schottky FEG and electron backscatter diffraction system (EBSD, Oxford Instruments) coupled to and INCA Aztec system (EDS, Oxford Instruments).

### 2.6. Hardness Studies

The hardness of the alloys was measured using an EMCO Test Duravision Vickers Hardness tester (Prufmaschinen GmbH, Kuchl, Austria). The machine was calibrated with a standard Vickers sample, before testing the samples. The samples were polished to grit of P400 with silicon carbide grinding paper (Struers). The applied mass for the hardness tester was set to 9.8 centinewton for all the alloys.

## 3. Results

### 3.1. Phase Calculations 

The phase transformation temperatures, as determined by differential scanning calorimetry, were 829 °C, 751 °C, 746 °C and 744 °C for the 1, 3, 5 and 10 wt.% Cu alloys, respectively. The measurements were in good agreement with the calculated values for the β-transus temperatures at 746 °C (10 wt.% Cu alloy) and 753 °C (5 wt.% Cu alloy). The discrepancy between the calculated and measured values increased at lower Cu additions. 

The thermodynamic prediction of phases for the alloys is given in Figure 1a,b as a function of temperature, and predicted HCP-Ti (α) and BCC-Ti (β), and additionally Ti_2_Cu for Cu additions of 1% and higher. At the heat treatment temperature of 747 °C, the calculated mol% of the phases is given in Table 5, where the 10 wt.% Cu alloy was predicted to have no α-Ti phase present, while alloys below 5 wt.% Cu were predicted to have no Ti_2_Cu phase present.

Given the prerequisites mentioned earlier, the Ti_2_Cu forms at 656 °C in the 1 wt.% Cu alloy (Figure 1a), which is below the annealing temperature. In addition, the predicted phase fraction at lower temperature is very small, thus the phase is not likely to nucleate on quenching due to kinetic reasons. 

### 3.2. XRD and Microstructure

**Figure 2:**
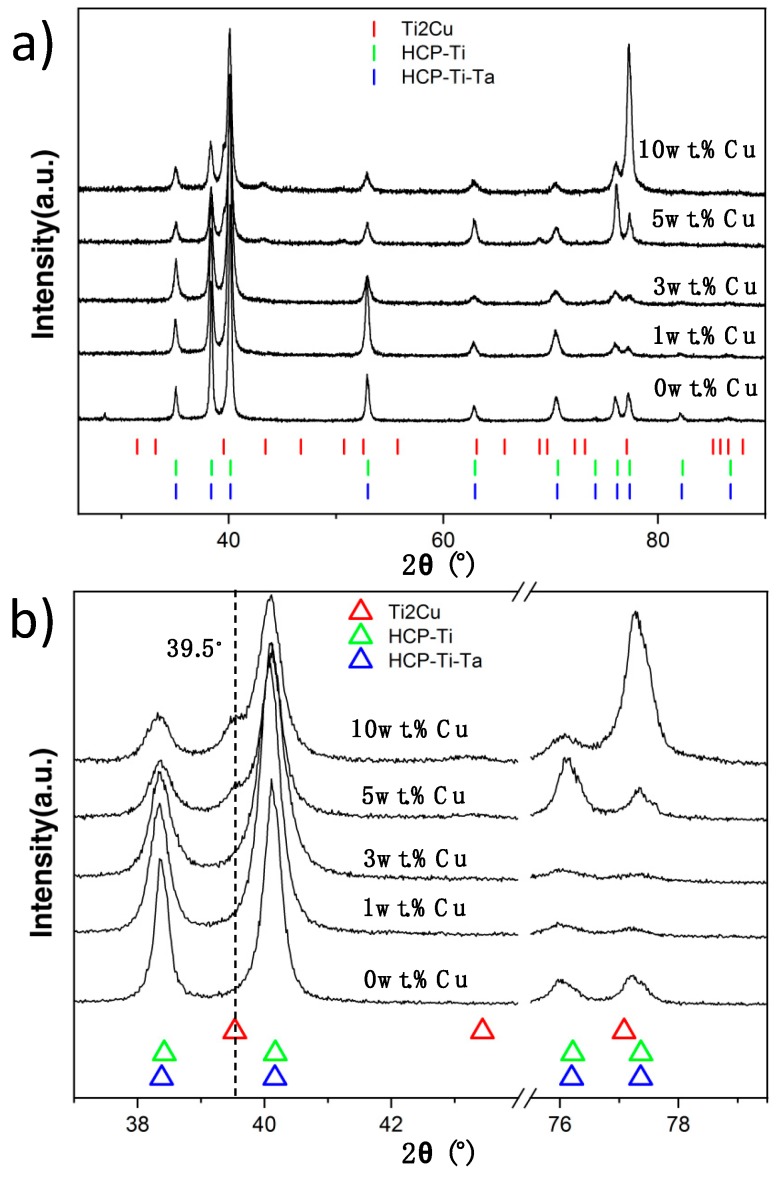
X-ray diffraction on TNTZ-Cu_x_ alloys: (**a**) Diffraction for all alloys including references from Ti_2_Cu (04-003-1382), HCP- (Ti-Ta) (03-065-9616) and HCP-Ti (00-044-1294). (**b**) X-ray diffraction pattern showing the 2θ angular ranges for the alloys from 38°–41° and 76°–78°. Note the Ti_2_Cu peak at 39.5°.

**Figure 3:**
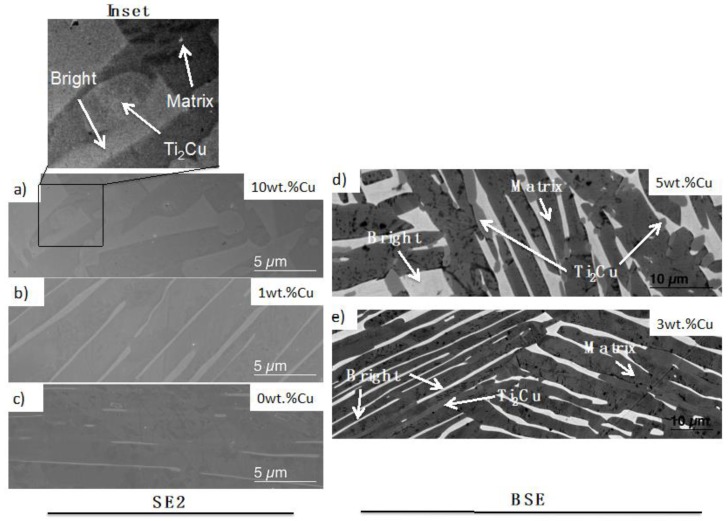
SEM micrographs showing (**a**) 10 wt.% Cu alloy with Inset showing three crystal phases, (**b**) 1 wt.% Cu alloy, (**c**) 0 wt.% Cu alloy (**d**) 5 wt.% Cu alloy with 3 crystal phases shown (**e**) 3 wt.% Cu alloy with three crystal phases shown.

With X-ray diffraction studies on the 0 wt.% Cu and 1 wt.% Cu alloys, only the α-phase was determined to be present (Figure 2). However, SEM imaging for the same alloys (Figure 3b,c) indicated that the materials had two crystal phases present, as predicted (i.e. within the α + β region). The bright phase is most likely remaining β-phase since Ta, Nb and Cu (where Cu was given by the calculations) are β-stabilisers and the heat treatment temperatures were at 747 °C, thus within the α-β region. 

In the 3 wt.% Cu alloy a third phase was predicted and observed in SEM imaging (Figure 3e). Diffraction peaks were not observed for this phase but it was assumed to be the predicted intermetallic Ti_2_Cu. However, a very low amount was observed at large grain boundaries (GBs), which was in line with the prediction. β-Ti was not detected in the X-ray diffractogram (Figure 2) for 3 wt.% Cu alloy either, but is present in SEM images (Figure 3e). When the Cu addition was increased, the microstructure became coarser grained with thicker and disrupted bright (β) phase lathes. This coarsening is observed when comparing the 3 wt.% Cu to the 5 wt.% Cu alloy (Figure 3e,d, respectively). Likewise the phase fraction of the bright Ta, Cu-rich phase increased from 3 to 5 wt.% Cu.

The 5 wt.% Cu alloy clearly had three phases, while only small precipitates were observed in the 3 wt.% Cu. The X-ray diffraction patterns displayed an increase in Ti_2_Cu phase with increase in Cu concentration from 5 wt.% Cu to 10 wt.% Cu (Figure 2b).

By comparing the 10 wt.% Cu and 5 wt.% Cu samples, it is clear that the microstructure is much coarser in the 10 wt.% Cu and that a higher volume fraction of the Ti_2_Cu phase is found in (Figure 3a and inset). The phase diagram for 10 wt.% Cu also predicts that the Ti_2_Cu phase exists, above the β-transus predicted to be at 747 °C and this is supported by the micrographs, which show large “globular” structures of the predicted Ti_2_Cu phase present (Figure 3a inset). These precipitates were found exclusively at the GBs between α-Ti and β-Ti, for all alloys (Figure 3). 

### 3.3. Chemical and Crystal Phase Analysis

A change in phase development with the precipitation of the Cu-rich phase (Ti_2_Cu) was observed in the 3 wt.% Cu and 5 wt.% Cu samples and thus they were the focus of further study. 

**Figure 4:**
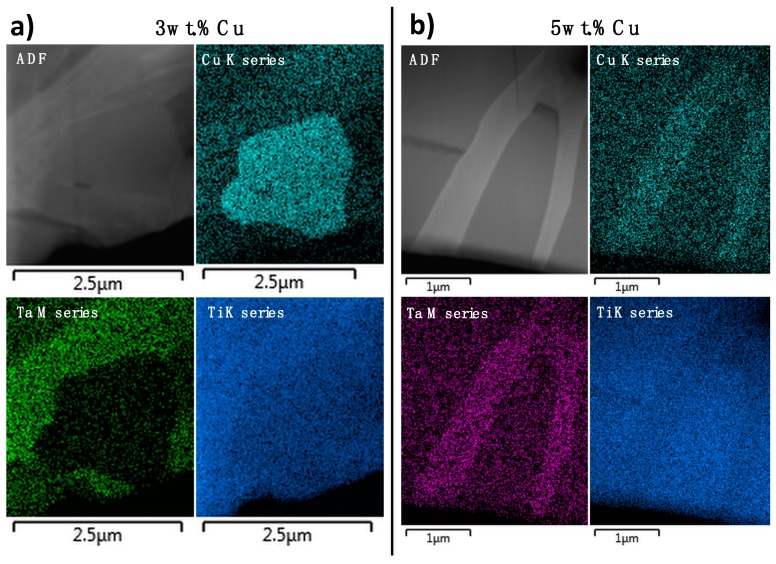
STEM-EDS maps on the crystal boundary showing 3 crystal phases for (**a**) 3 wt.% Cu alloy and (**b**) 5 wt.% Cu alloy, with associated Annular dark field detector image, Cu K series map, Ta M series map and Ti K series map.

**Figure 5:**
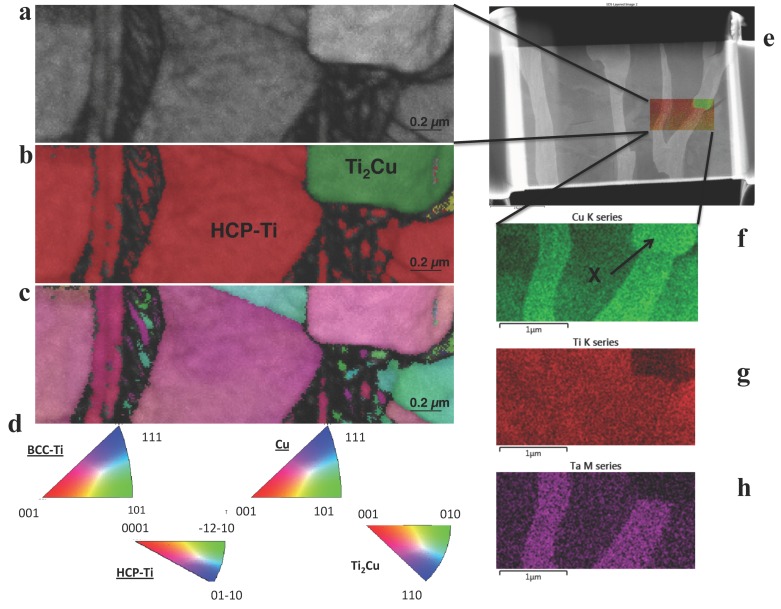
3 wt.% Cu alloy studied using TKD and EDS. From the TKD study (**a**) displays band contrast, (**b**) a phase map and (**c**) IPF Z map of the same area with (**d**) associated pole figures. (**e**) An electron image of the area investigated with both techniques, (**f–h**) EDS maps of: (**f**) Cu K series (where X indicates Ti_2_Cu), (**g**) Ti K series and (**h**) Ta M series.

The 3 wt.% Cu alloy had a microstructure similar to the lower Cu content alloys with thin lathes, but by using backscattered electron imaging, smaller precipitates were discovered at the GBs of the larger α-Ti grains (Figure 3e). These areas were studied further by preparation of focused ion beam (FIB) lamella, STEM-EDS and transmission Kikuchi diffraction (TKD). Regions of Cu-rich precipitates were observed, with adjacent crystals containing Ti and Ta (Figure 4a). The grains with the brightest contrast, which probably was β-Ti considering the heat treatment temperature, were also slightly coarser grained in the 3 wt.% Cu alloy compared to those with lower Cu content. The phases were assigned as a matrix phase of α-Ti, Ti_2_Cu and a bright phase, where the bright phase could not be assigned to a known crystal phase using TKD (Figure 5). The 5 wt.% Cu alloy had a microstructure of irregular lathes compared to those with lower Cu content (Figure 3). The lathes that formed were not straight-line structures as in the 3 wt.% Cu, but instead lathes disrupted by Cu-rich globules, formed along the length of the bright β-phase. 

**Figure 6:**
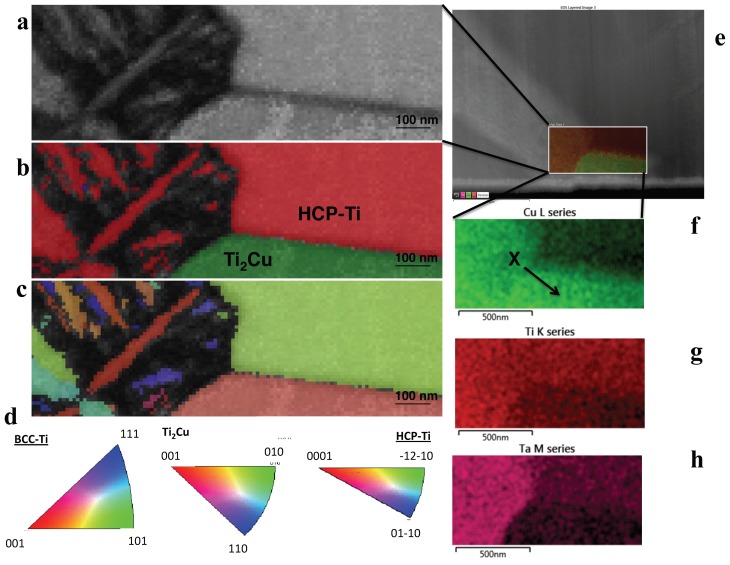
5 wt.% Cu alloy studied using TKD and EDS. From the TKD study (**a**) displays band contrast, (**b**) a phase map and (**c**) IPF Z map of the same area with (**d**) associated pole figures. (**e**) An electron image of the area investigated with both techniques, (**f–h**) EDS maps of: (**f**) Cu L series (where X indicates Ti_2_Cu), (**g**) Ti K series and (**h**) Ta M series.

Using TKD, the Cu-rich phase and the matrix phase were designated as Ti_2_Cu and α (HCP-Ti), respectively (Figure 6). Assignment of a known crystal to the bright phase was challenging using TKD for this alloy as well (Figure 6). The formation of the Cu-rich phase- in 3 wt.% Cu and 5 wt.% Cu occurred selectively at the GBs between the α-Ti and the bright phase. Spectroscopic comparison of the 3 wt.% Cu and 5 wt.% Cu showed that the former alloy contained a crystal with more Cu in a “globular” shaped crystal, surrounded by a crystal with more Ta and Ti (Figure 4a). The 5 wt.% Cu contained a thin crystal enriched with Cu and Ta and surrounded by crystals of Ti with lower concentrations of Cu with Ta (Figure 4b). Using TKD coupled to EDX mapping on a different lamella, the 3 wt.% Cu alloy showed a bright phase crystal with Cu, Ta and Ti (Figure 5f–h).

### 3.4. Hardness

**Figure 7:**
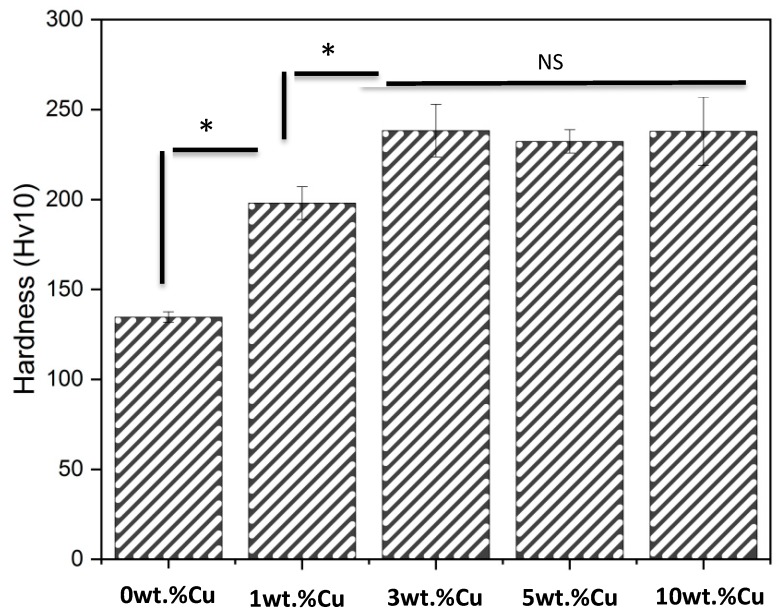
Vickers hardness of the TNTZ-Cu_x_ alloys (“*” indicates statistical significance of p < 0.05, as per Tukey Anova test, while “NS” indicates no statistically significant difference).

The hardness (Figure 7) of the 0 wt.% Cu alloy (135 ± 3 Hv) was significantly lower than the 1 wt.% Cu alloy (198 ± 9 Hv, p = 0.0005), which in turn was significantly lower than the 3 wt.% Cu, 5 wt.% Cu and 10 wt.% Cu alloys (p < 0.035). No statistically significant difference in hardness was found among the 3 wt.% Cu (238 ± 15 Hv), 5 wt.% Cu (232 ± 7 Hv) and 10 wt.% Cu (238 ± 19 Hv) alloys (p > 0.975).

## 4. Discussion

The present study investigated the addition of copper to a TNTZ alloy and its effect on the microstructure, with the scope of developing a biomedical alloy with potential antibacterial ability in future. Predictions of the stable phases in the alloy system - that are to be regarded as an initial approach to the development of TNTZ-Cu_x_ alloys-revealed 3-phases in equilibrium (Figure 1).

Comparison of the predicted β-transus temperatures to the experimentally observed values, revealed discrepancies in the data at 829 °C, 751 °C, 746 °C and 744 °C for the 1, 3, 5 and 10 wt.% Cu alloys, respectively. The measurements were in good agreement with the calculated values of 746 °C and 753 °C, for the 10 wt.% Cu and 5 wt.% Cu alloys. However, the discrepancy between the calculated and measured values increased as the Cu content decreased. The reason for the discrepancies could be the absence of the Ti-Ta-Cu system in the database. An additional cause for variance in the discrepancies could be due to the reduction in the effective Cu content, since Cu is bonded in the intermetallic (Ti_2_Cu) phase, which was identified by diffraction for the 5 and 10 wt.% Cu alloys. A further reason could be that the β-stabilizers of Ta and Nb are soluble in the intermetallic phases, in addition to Cu. It is also uncertain whether the metastable Ti_3_Cu [24] is present for the lower Cu compositions, thus further research is required. 

The changing copper concentrations in the TNTZ materials also changed the phase development by affecting the “driving force” (change in Gibb’s free energy), leading to one of three phase development scenarios (Table 2). For the 5 wt.% Cu alloy, the β- and Ti_2_Cu-transus temperatures are within 1 °C of each other and could lead to precipitation of phases according to $\beta \;\to \;Ti_{2}Cu+\alpha$. For Cu concentrations below 5 wt.% Cu, the phase development will likely proceed according to according to the reaction $\Delta G_{Ti2Cu}>\Delta G_{\alpha }$. When the Cu concentration is below the 5 wt.% Cu, the phase development will likely proceed according to the reaction $\Delta G_{Ti2Cu}<\;\Delta G_{\alpha }$. Cu addition also affects the volume fraction of the phases that develop and thermodynamic modelling predicted that no α-phase would nucleate for the 10 wt.% Cu alloy quenched from 747 °C (Table 5). Predictions were also made that no Ti_2_Cu would nucleate in the quenched 3 wt.% Cu alloy (Table 5). A possible reason for this difference is that the quenching rates from 747 °C, might have been too slow, and thus the α-phase nucleated in the 10 wt.% Cu alloy. The same process could have caused the Ti_2_Cu to nucleate in the 3 wt.% Cu alloy. Alternatively, deviations in the furnace temperature prior to quenching might also be responsible for the nucleation of the phases. Further investigations in modelling and rapid quenching could elucidate the cause for the differences between predictions and experiments.

The 0 wt.% Cu TNTZ was heat treated at 747 °C, and predicted to have a microstructure consisting of α (76.4%) and β (23.6%), with a hardness of 135 ± 3 Hv. In a previous study [11] the alloy was found to be a α (50%) and β (50%) alloy with hardness of 340 HVN. The differences found were probably due to various forging treatments of the alloy in the previous study [11]. The addition of 1 wt.% Cu did not cause a third phase to precipitate, presumably due to the fact that the Ti_2_Cu phase only forms at temperatures lower than 747 °C (Figure 1a). Therefore the 0 wt.% Cu and 1 wt.% Cu alloys are confirmed as two-phased materials via diffraction (Figure 2) and microscopy studies (Figure 3).

The 3 and 5 wt.% Cu alloys both had a 3-phased (Figure 3d,e) crystal structure, even though calculation of phases at 747 °C predicts a 2-phase structure (α and β) for the 3 wt.% Cu (Table 5). This indicates that precipitation could have taken place below 747 °C prior to the quenching into salt water. Alternatively, since 3 wt.% Cu is lower than the 5 wt.% Cu, the precipitation of α might have occurred according to the reaction $\beta +\alpha \to \;\alpha +Ti_{2}Cu$, which is in line with kinetics for active eutectoid transformations described in studies on Ti-Cu [18,19]. The Τi_2_Cu was not observed in the XRD pattern of the 3 wt.% Cu but this could be due to the volume fraction being below 2 wt.% of Τi_2_Cu, which is the detection limit for the X-ray diffraction technique [20]. The 5 wt.% Cu however showed the Τi_2_Cu in the diffractogram (Figure 2) and these were present as “globular” and irregular crystals in the microstructure. The bright phase was observed as “globular” shaped in the 5 and 10 wt.% Cu alloys. 

The standard crystal structures assigned using TKD were HCP-Ti [29] and Τi_2_Cu [30], since these crystals gave the most appropriate match to the experimental data. The indexing of 3- and 5 wt.% Cu in TKD gave similar chemical phases of the Ti-Ta (bright β phase), the α (HCP-Ti) phase as well as (Τi_2_Cu) phase. The β phase was nano-crystalline and therefore contained many internal GBs, resulting in difficulty for the computational phase assignment routines in TKD to assign these GB pixels to specific phases [31], resulting in an inability to index the crystals. The α (HCP-Ti) and Τi_2_Cu however were indexed and matched well to the standard crystals. EDX-maps of the crystals in SEM (Figure 5 and Figure 6) and STEM (Figure 4) provide a view to the “globular” structure of the Cu-rich phases in the 5 wt.% Cu, while lathes of Cu-rich crystals were discerned in the 3 wt.% Cu (Figure 3e). This difference in the structure for the Cu-rich phase could be due to particle coarsening, at higher Cu concentrations, via diffusion in the matrix of the alloy [32]. The maps also indicated that the 5 wt.% Cu had thin crystals containing Ta and Cu with Ti in adjacent phases (Figure 4b). The thin crystal could be the bright phase enriched in Ta surrounded by the Ti-rich α-Ti matrix. The 3 wt.% Cu had crystals enriched in Cu (Figure 4a) with Ta surrounding the crystals. These Cu-rich crystals could be the Ti_2_Cu phase that was surrounded by the Ta-rich bright phase found in the study. 

The 10 wt.% Cu alloy had a 3-phased microstructure (Figure 3) with α, Τi_2_Cu and β (bright) phase, which contradicts thermodynamic modelling predictions, where the α phase was calculated as absent (Table 5). The prediction of only β and Τi_2_Cu after a rapid quench is not experimentally observed in microstructural transformations of active β-eutectoid alloys, of which Ti-Cu is one such example [18,33]. This microstructure contains “globular” precipitates of a Cu-rich phase (Ti_2_Cu) that seems to form between the α and β phase (Figure 3a inset), similar to 3- and 5 wt.% Cu. The bright (β) phase seems to have changed to a “globular” shaped structure with the increase in wt.% Cu, similar in shape to the matrix (α) phases. While the 10 wt.% Cu was not studied in TKD, it is probable that the β (bright) phase would index the same as in the 3- and 5 wt.% Cu. All transformation reactions in these alloys containing the bright phase seem to preclude β phase formation, and it is possible that the reason could be martensitic phase transformations, on account of the rapid cooling [34]. 

The Cu addition to the TNTZ material drives the alloy to rapid transformations via the eutectoid reactions. For the 5 wt.% Cu and 3 wt.% Cu, the bright phase could not be indexed as α, and might be a martensitic phase. This is observed in Ti-Ta (1–10 at.% Ta) and the phase identified was orthorhombic $\alpha^{\prime \prime }$ [34] according to the reaction $\beta \to \alpha^{\prime \prime }\to \alpha$. This reaction is thought to occur exclusively at temperatures below 927 °C (1200 K) and involves mechanical shearing and shuffling of atoms to achieve the transformation [35]. The exact shearing planes are however presently being debated and could involve twinning shear in one or both of the planes of $\left\{332\right\}11\bar{3}$ [36] and 111{112} [37].

The hardness increased significantly with the addition of Cu (Figure 7). This could be a consequence of the Cu atoms dissolving into the α and β crystal phases as a solid solution mixture. The hardness of the 3 wt.% Cu, 5 wt.% Cu and 10 wt.% Cu was not significantly different but was significantly higher than 0 wt.% Cu and 1 wt.% Cu, which could indicate that the saturation limit of solid solution alloying had been reached. 

## 5. Conclusions

In the present study, the effect of Cu addition to an existing TNTZ alloy was investigated. The alloys in the range of 3 to 10 wt.% Cu all had Ti_2_Cu present, while in alloys with less than 3 wt.% Cu, Ti_2_Cu was not observed. From 5 to 10 wt.% Cu, the alloys showed the presence of Ti_2_Cu in increasingly “globular” structures with increase in Cu concentration. An associated effect of increasing to Cu content from 0 to 3 wt.% Cu was that the hardness increased, but no additional increase was achieved from 3 to 10 wt.% Cu. The hardness could be a result of solid solution strengthening, but might also be affected by martensitic transformations. While the material has reasonable hardness, the potential antibacterial ability of the material requires assessment in future. Therefore further studies are envisioned for this alloy system to optimize the mechanical, antibacterial and corrosion properties for the purpose of producing a suitable antibacterial implant material.

## Figures and Tables

**Table 1:** Calculated β- and Ti_2_Cu-transus temperatures for the investigated alloys.

Cu Addition [wt.%]	Calc. β-transus [Celsius]	Calc. Ti_2_Cu-transus [Celsius]
0	814	N/A
1	802	655
3	777	728
5	753	752
10	746	859

**Table 2:** Reaction equations for stable phases and microstructural development.

$\Delta G_{Ti_{2}Cu}\;<\;\Delta G_{\alpha }$	$\Delta G_{Ti_{2}Cu}\;=\;\Delta G_{\alpha }$	$\Delta G_{Ti_{2}Cu}\;>\;\Delta G_{\alpha }$
β →α $\beta +\alpha \to \;\alpha +Ti_{2}Cu$	$\beta \;\to \;Ti_{2}Cu+\alpha$	$\beta \;\to \;Ti_{2}Cu$ $\beta +Ti_{2}Cu\;\to Ti_{2}Cu+\;\alpha$

**Table 3:** Nominal weight percentages of elements in the TNTZ-Cu_x_ alloys.

Designation	Cu	Ti	Nb	Zr	Ta
0 Cu	0	86.6	1.7	1.6	10.1
1 Cu	1	85.74	1.68	1.58	10.0
3 Cu	3	84	1.65	1.55	9.8
5 Cu	5	82.26	1.62	1.52	9.6
10 Cu	10	77.94	1.53	1.44	9.09

**Table 4:** The 3-step metallographic preparation of TNTZ-Cu_x_. All products sourced from Struers, except H_2_O_2_, which was sourced from BASF SE (Ludwigshafen, Germany).

Steps	1–Grind	2–Rough Polish	3–Final Polish
Surface	SiC–120 P	MD–Dur cloth	MD–Floc cloth
Abrasive	-	6 µm diamond suspension	OP-S Si-Colloids and H_2_O_2_ (5:1) solution
Lubricant	water	DP lubricant red	-
Speed (rpm)	250 contra	200 contra	150 contra
Duration (min)	until planar	30 min	30 min

**Table 5:** Calculated mol% of phases of α, β and Ti_2_Cu in the investigated alloys at 747 °C.

	Calculated Phases [mol%]		
Cu Addition [wt.%]	α	β	Ti_2_Cu
0	76	24	0
1	37	63	0
3	35	65	0
5	14	85	2
10	0	85	15
